# Increased expression of the receptor for advanced glycation end-products in human peripheral neuropathies

**DOI:** 10.1002/brb3.176

**Published:** 2013-10-08

**Authors:** Judyta K Juranek, Pratik Kothary, Alka Mehra, Arthur Hays, Thomas H Brannagan, Ann Marie Schmidt

**Affiliations:** 1 Department of Surgery, Columbia University Medical CenterNew York, New York; 2 Diabetes Research Program, Department of Medicine, NYU Medical CenterNew York, New York; 3 Infectious Diseases Center, Department of Medicine, NYU Medical CenterNew York, New York; 4 Department of Pathology, Columbia University Medical CenterNew York, New York; 5 Department of Neurology, Columbia University Medical CenterNew York, New York

**Keywords:** Diabetes, human, peripheral neuropathy, RAGE, RAGE ligands

## Abstract

**Background:**

Diabetic neuropathy and idiopathic neuropathy are among the most prevalent neuropathies in human patients. The molecular mechanism underlying pathological changes observed in the affected nerve remains unclear but one candidate molecule, the receptor for advanced glycation end-products (RAGE), has recently gained attention as a potential contributor to neuropathy. Our previous studies revealed that RAGE expression is higher in porcine and murine diabetic nerve, contributing to the inflammatory mechanisms leading to diabetic neuropathy. Here, for the first time, we focused on the expression of RAGE in human peripheral nerve.

**Methods:**

Our study utilized de-identified human sural nerve surplus obtained from 5 non-neuropathic patients (control group), 6 patients with long-term mild-to-moderate diabetic neuropathy (diabetic group) and 5 patients with mild-to-moderate peripheral neuropathy of unknown etiology (idiopathic group). By using immunofluorescent staining and protein immunoblotting we studied the expression and colocalization patterns of RAGE and its ligands: carboxymethyllysine (CML), high mobility group box 1 (HMBG1) and mammalian Diaphanous 1 (mDia1) in control and neuropathic nerves.

**Results:**

We found that in a normal, healthy human nerve, RAGE is expressed in almost 30% of all nerve fibers and that number is higher in pathological states such as peripheral neuropathy. We established that the levels of RAGE and its pro-inflammatory ligands, CML and HMBG1, are higher in both idiopathic and diabetic nerve, while the expression of the RAGE cytoplasmic domain-binding partner, mDia1 is similar among control, diabetic, and idiopathic nerve. The highest number of double stained nerve fibers was noted for RAGE and CML: ∼76% (control), ∼91% (idiopathic) and ∼82% (diabetic) respectively.

**Conclusions:**

Our data suggest roles for RAGE and its inflammatory ligands in human peripheral neuropathies and lay the foundation for further, more detailed and clinically oriented investigation involving these proteins and their roles in disorders of the human peripheral nerve.

## Introduction

Diabetic neuropathy and idiopathic neuropathy are among the most prevalent neuropathies affecting the peripheral nerve in human subjects (Dyck et al. 1981; Barohn 1998).

Regardless of their etiology, it is conceivable that the molecular mechanisms underlying pathological changes observed in the affected nerve might share common features with neuropathies secondary to known etiologies, such as diabetes. One such potential multiaction protein contributing to the pathogenesis of neuropathy may be the receptor for advanced glycation end-products (RAGE). RAGE is a multiligand receptor of the cell surface immunoglobulin superfamily involved in inflammatory responses, oxidative stress, and cellular dysfunction in a number of conditions and diseases (Schmidt et al. 2000; Bierhaus et al. 2005). In the last decade, a growing number of studies revealed that RAGE may play a role in central nervous system (CNS) neurodegenerative disorders such as Alzheimer's disease, Parkinson's disease, Creutzfeldt–Jakob’ disease, and Huntington's disease (Brenn et al. 2011; Anzilotti et al. 2012; Teismann et al. 2012) and peripheral neuropathies such as familial amyloid polyneuropathy (Sousa and Saraiva 2003; Bierhaus et al. 2004; Haslbeck et al. 2005), Charcot neuroarthropathy (Witzke et al. 2011), vasculitic neuropathy (Haslbeck et al. 2004), and especially diabetic neuropathy (Bierhaus et al. 2004; Haslbeck et al. 2005; Toth et al. 2008). Recently, we have shown that the level of RAGE is higher in the peripheral nerve of the hyperglycemic versus control nondiabetic pig (Juranek et al. 2010) and might contribute to the development of diabetic neuropathy by enhancing macrophage responses and polarization in the murine diabetic nerve subjected to acute nerve crush (Juranek et al. 2013). Though the detailed mechanism by which RAGE executes its actions and exacerbates existing neuropathological conditions remains under investigation, emerging evidence suggests that the mechanism triggering RAGE-related neurodegenerative processes is likely related to oxidative stress, increased production of advanced glycation end-products (AGE) and their binding to RAGE and subsequent RAGE-dependent activation of downstream factors, such as the NF-κB inflammatory pathway (Schmidt et al. 1996; Haslbeck et al. 2007). Carboxymethyllysine (CML), one of the most prevalent AGEs in vivo, is considered to be a marker of oxidative stress and cellular damage (Ramasamy et al. 2007; Sugimoto et al. 2008) and a potential contributor to neuropathic changes in the peripheral nerve (Schmidt et al. 1996; Sugimoto et al. 1997; Haslbeck et al. 2002; Kawai et al. 2010). Apart from pro-inflammatory AGE binding, RAGE interacts with distinct proteins, among them high mobility group box 1 (HMGB1). HMGB1, a pro-inflammatory protein that may function as a neuromodulatory cytokine after tissue damage or injury (Faraco et al. 2007; Feldman et al. 2012), has recently been shown to play a role in maintenance of neuropathic pain behavior in rodents (Feldman et al. 2012), mediation of ischemic brain damage via RAGE binding (Muhammad et al. 2008) and contribution to pain hypersensitivity after peripheral nerve injury (Shibasaki et al. 2010). In the intracellular space, the RAGE cytoplasmic domain binds to mammalian Diaphanous 1 (mDia1) (Hudson et al. 2008). mDia1 belongs to a multidomain formin family involved in actin and microtubule remodeling (Baarlink et al. 2010; Goh et al. 2012) and it has been recently shown to contribute to RAGE-stimulated cell proliferation/migration in ligand-stimulated smooth muscle cells.

In the present work, we studied the expression of RAGE in the peripheral nerve fibers and its colocalization with ligands, CML, HMGB1, and mDia1 in three different human peripheral nerve conditions. Our goal was to establish morphological evidence of RAGE and its ligands in the peripheral nerve and lay the foundation for further, more detailed and clinically oriented investigation involving these proteins and their roles in disorders of the human peripheral nerve.

## Materials and Methods

The study group consisted of six male patients of mean age 62.5 years (range 41–86) with long-term (6–20 year range) diabetes mellitus and progressive mild-to-moderate peripheral neuropathy as determined by clinical examination, electrophysiological tests, and histopathological examination of tissue samples. Additionally, five male patients of mean age 74.5 years (range 61–90 years) with mild-to-moderate neuropathy of unknown etiology and five age-matched control subjects identified from the neuromuscular pathology laboratory at Columbia University Medical Center, who did not have diabetes or neuropathy but had other diagnoses such as myopathy.

Nerve biopsies from diabetic patients were previously obtained for clinical care following standard procedures (Younger et al. 1996). Each patient underwent a full-thickness open biopsy of the sural nerve under local anesthesia through a vertical incision centered approximately 12 cm above the lateral malleolus. This study on human nerves utilized surplus deidentified tissue obtained from biopsy and was approved by the Columbia University Institutional Review board.

### Immunofluorescence

After retrieval, human specimens were immediately placed into and stored in the isopentane-liquid nitrogen container for further processing. Frozen samples were mounted in optimal cutting temperature compound (Tissue-Tek O.C.T.; Sakura Finetek, Zoeterwoude, Netherlands), cut transversely and longitudinally at 10 μm thickness on a cryostat (Microm HM 550; Thermo Scientific, Waltham, MA) and collected on polylysine-coated slides (SuperFrost Plus; Fisher Scientific, Pittsburgh, PA). After collection, specimens were fixed for 5 min in cold acetone sections and then processed following the standard immunostaining protocol. Briefly, all specimens were allowed to dry for 1–2 h at room temperature, incubated with blocking solution (Cas-block; Invitrogen, Grand Island, NY) for 1 h, and incubated overnight with primary antibodies: goat anti-human RAGE (1:100, GTX27764; Genetex, Irivine, CA) for single staining or mix of antibodies: (1) fiber staining: goat anti-RAGE antibody and chicken anti-neurofilament (1:200, heavy chain, ab72996; Abcam, MA) (2) ligand colocalization – goat anti-RAGE and rabbit anti-CML (ab27684), anti-HMGB1 (ab18256), or anti-mDia1 (ab11172) (all antibodies, 1:100; Abcam, MA). The next day, sections were rinsed 4 × 5 min in phosphate buffered saline (PBS), incubated with secondary antibodies: chicken anti-goat Alexa 594 and goat anti-chicken or goat anti-rabbit Alexa 488 (1:300 and 1:200, respectively; Invitrogen), for 1 h, rinsed again 4 × 5 min in PBS and mounted in fluorescent mounting medium (DAKO-Invitrogen). To control for specificity of secondary antibodies and to minimize risk of false positive results, standard immunostaining procedures with omission or replacement of primary antibodies on sections from each tissue sample set was carried out parallel to the experimental staining. Mounted sections were examined with Zeiss AxioVision (Zeiss, Goettingen, Germany) and Leica SP5 scanning confocal microscope (Leica SP5, Goettingen, Germany) at 20× and 40× objective magnification. Quantification of RAGE-positive fibers, from single stained cross and longitudinal control and neuropathic nerve tissues and colocalization analysis of double stained sections was performed on 200 μm^2^ regions of interest (ROI) as previously described (Juranek et al. 2013). Number of single and double stained fibers was calculated using ImageJ NIH open source software (http://rsb.info.nih.gov/ij/), ImageJ Cell Counter and Colocalization plugin, respectively, following Image J guidelines; control nerve values were used as a reference (100% of all positive fibers). Signal intensity ratio was quantified using ImageJ Analyze tool, mean pixel intensities for a given ROI were compared, the control group was used as a reference.

All values are presented as mean ± standard error (SEM). The statistical significance of differences (*P* < 0.05) was evaluated by nonparametric analyses of variance (ANOVA) and two-tailed *t*-test (GraphPad Instat, CA).

### Immunoblotting

Snap frozen, nonfixed control, and neuropathic nerve samples were pooled (*n* = 3 per condition) and homogenized in chilled tissue extraction buffer (Invitrogen) using Kontes tissue grinders (Kimble Chase, Vineland, NJ). A small aliquot was kept for protein estimation and the remaining portion was frozen for further processing. Protein electrophoresis was carried out using X Cell II™ Blot Module (Invitrogen) following protocols supplied by the manufacturer. Briefly, 40 μg protein samples were loaded on NuPAGE® Novex® Bis-Tris 4–12% gradient gel (Invitrogen), run at 200 V for 50 min, transferred to nitrocellulose membranes at 30 V for 1 h, blocked in 5% milk (Sigma, St. Louis, MO) for 1 h, incubated with goat anti-RAGE (Genetex) at dilution 1:1000, at 4°C overnight, washed with TBST 4 × 5 min (Tris-buffered saline with 0.1% Tween 20), incubated with horseradish peroxide (HRP)-conjugated anti-goat antibody for 1 h, washed with TBST 4 × 5 min and developed using enhanced chemoluminescence (ECL) reagents (GlaxoSmithKlein, Uxbridge, U.K.). To verify loading amounts, membranes were stripped and reprobed with anti-GAPDH immunoglobin G (1:1000; Genetex) and used as a reference for relative blot density quantification (Image J, gel analysis).

## Results

### RAGE distribution in neuronal fibers of human sural nerve

**Figure 1:**
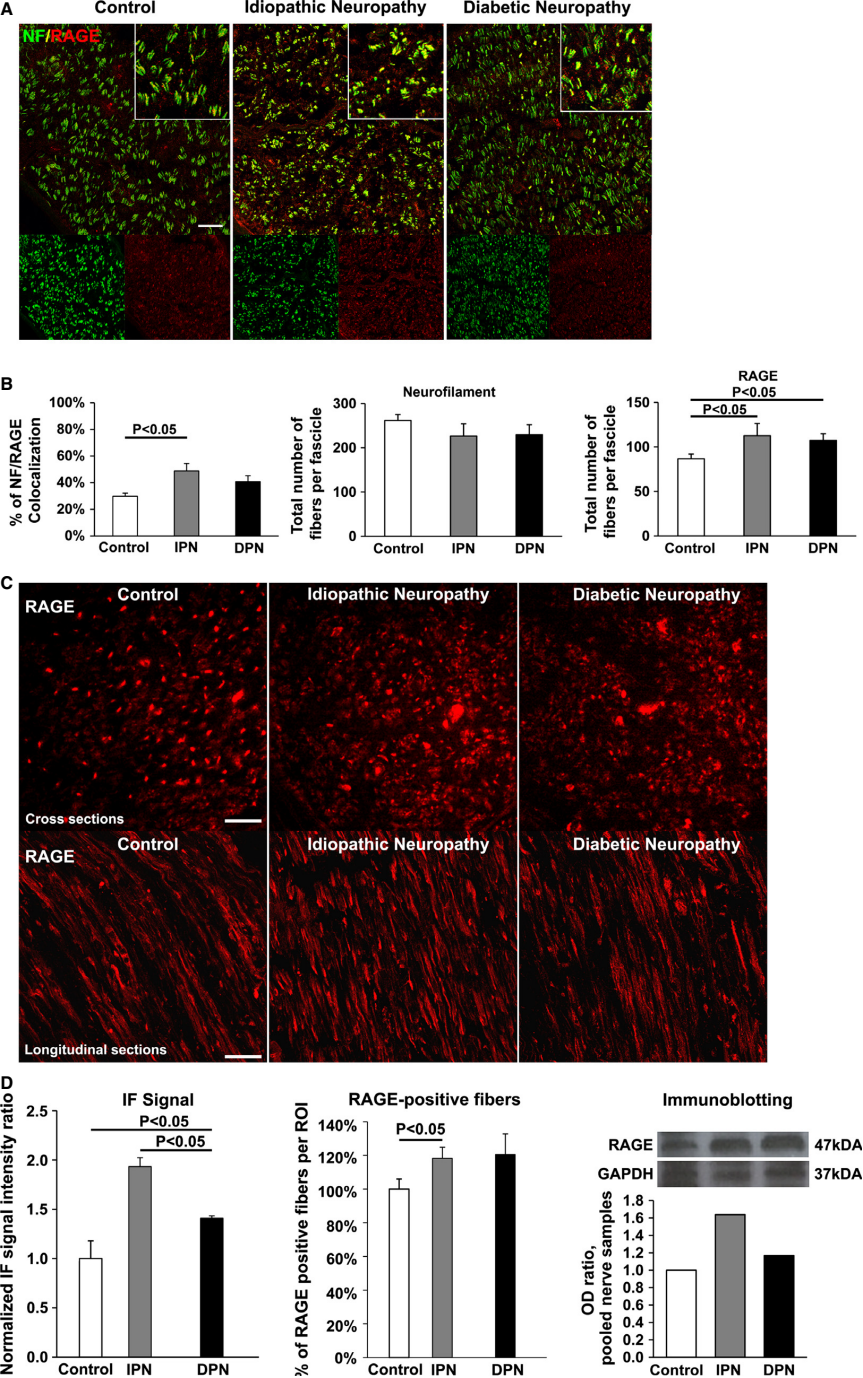
RAGE expression in human peripheral nerve fibers. (A) Neurofilament (NF, green)/RAGE (red) colocalization in normal (control), idiopathic (IPN), and diabetic (DPN) nerve, scale bar = 50 μm. Higher magnification insets show higher NF/RAGE colocalization in neuropathic groups, (B) Statistical difference in the number of double stained NF/RAGE fibers is observed between control and idiopathic nerve, *n* = 4 per each condition. Total number of NF- and RAGE-positive fibers per fascicle was calculated and statistical differences were observed for RAGE between groups, (C) Difference in staining intensities and number of RAGE-positive points are noted in both cross and longitudinal sections. Statistical analysis of the staining quantification, (D) revealed significant differences in the normalized immunofluorescent signal intensity ratio and the percentage of RAGE-positive fibers as compared to the control, *n* = 5 per each condition, scale bar = 50 μm, and (E) immunoblotting analysis of pooled (*n* = 3 per each condition) nerve homogenates supported immunostaining results. Values shown are the ratio of RAGE/GAPDH intensities. OD, optical density.

We began our study by investigating the neuronal expression of RAGE in peripheral nerve fibers of the control and neuropathic sural nerve (Fig. 1A and B) by colocalizing it with neuronal marker, neurofilament (NF). Our results revealed that in the control nerve, ∼29.8 ± 2.5% of all NF-positive fibers stained for RAGE, whereas in the idiopathic and diabetic nerve, the number of all NF-positive fibers stained for RAGE was higher, ∼48.9 ± 5.5% and ∼40.8 ± 4.4, respectively. There was a statistical difference in the number of double stained NF/RAGE fibers between the control and idiopathic nerve. The total number of NF-positive fibers per group was as follows: control – 261.7 ± 13.4, idiopathic – 226.7 ± 27.5, diabetic – 229.4 ± 22.75. There was no significant difference between groups. The total number of RAGE-positive fibers per group was as follows: control – 86.7 ± 5.4, idiopathic – 112.7 ± 13.8, diabetic – 107.3 ± 7.5. There were significant differences (*P* < 0.05) between control group and both neuropathic groups, but there was no difference within neuropathic groups.

### RAGE expression is higher in the idiopathic and diabetic neuropathic nerve

After establishing the pattern of neuronal expression of RAGE in the peripheral nerve under the control, idiopathic, and diabetic conditions, we conducted a comparative study using values from control nerve as a reference. We found that the immunofluorescence intensity and the number of RAGE-positive fibers were significantly higher for both idiopathic and diabetic neuropathy as compared to the healthy, control nerve. There was no statistically significant difference between the neuropathic nerves. However, there was a trend toward higher RAGE expression in the idiopathic nerve (Fig. 1C and D). Western blot data supported the immunostaining results. Optical density ratio of RAGE/GAPDH was determined and RAGE/GAPDH in the control nerve was set as reference (Fig. 1E).

### RAGE and its ligands, comparative analysis

**Figure 2:**
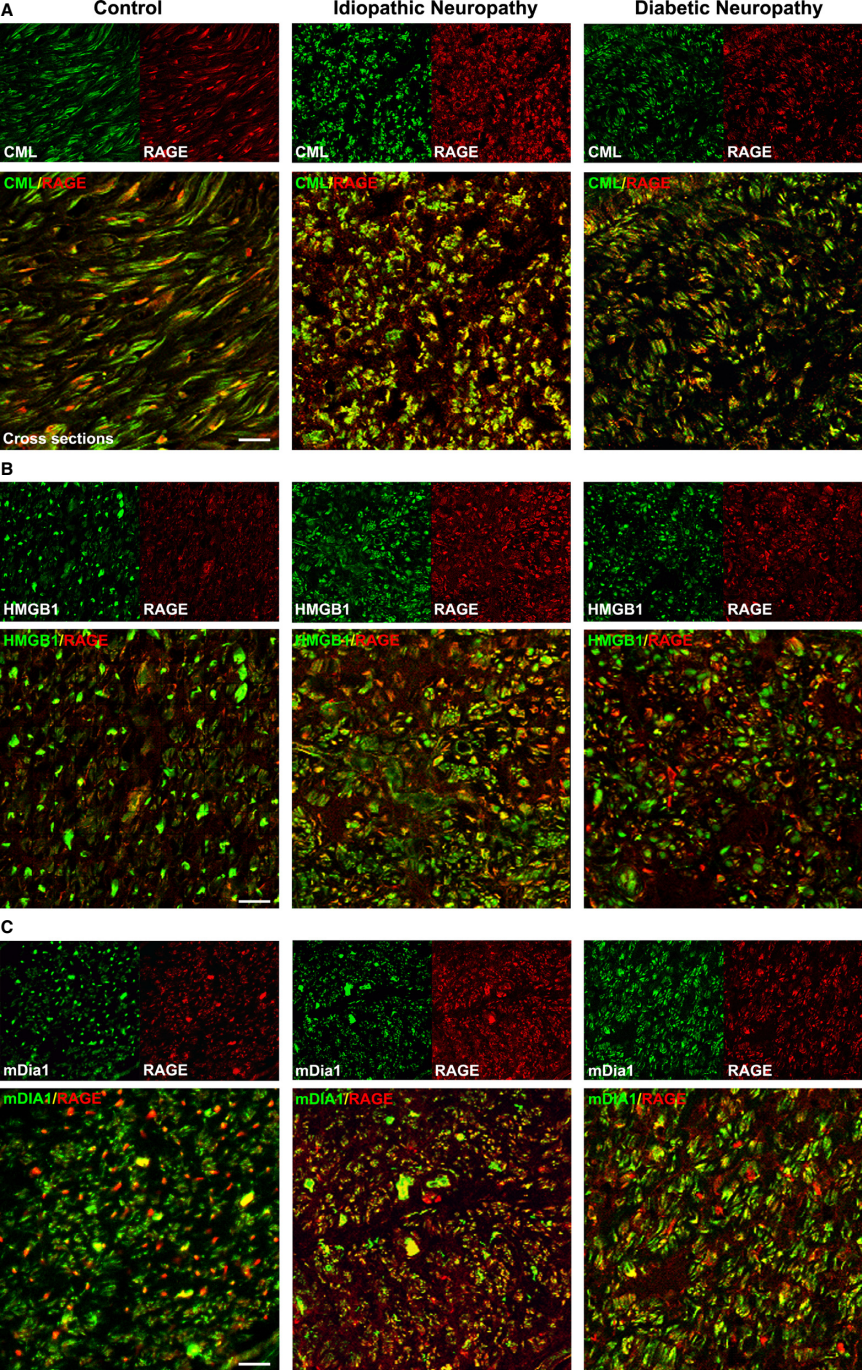
Expression of RAGE and its ligands in human nerve. RAGE (red) – CML (A, green), HMGB1 (B, green), and mDia1 (C, green) expression and colocalization study, *n* = 5 per each condition, scale bar = 50 μm.

**Figure 3:**
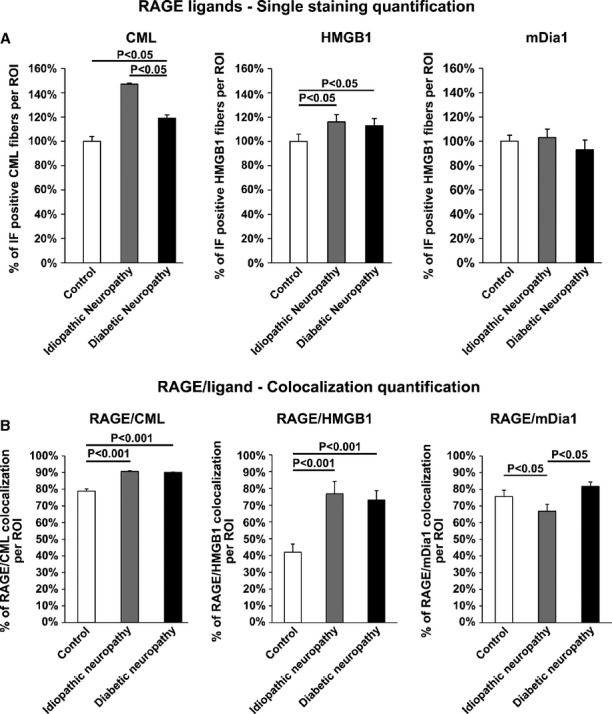
Statistical analysis of RAGE – ligand expression in human nerve. (A) CML, HMGB1, and mDia1 single staining quantification. Statistical differences between control/idiopathic (IPN) and idiopathic/diabetic (DPN) nerve were observed for CML and between control and both neuropathic nerves for HMGB1. No statistical difference was noticed for mDia1. (B) Number of RAGE fibers expressing CML and HMGB1 was statistically significantly higher in neuropathic nerves as compared to the control. On the contrary, the number of RAGE fibers costained for mDia1 in the idiopathic nerve were significantly lower than that in the control nerve, and there were significant differences between idiopathic (lower) and diabetic nerves, *n* = 5 per condition.

Next, we investigated the expression of three most prominent RAGE ligands: CML, HMGB1, and mDia1 (Figs. 2 and 3). The highest, statistically significant difference between control and neuropathic nerve was observed for CML, one of the specific AGE molecules. HMGB1 staining revealed higher and statistically significant differences in expression in both diabetic and neuropathic nerves versus control nerve. Finally, mDia1 staining showed no difference in expression in the idiopathic nerve and a trend toward lower levels in the diabetic nerve, but no change was statistically significant (Fig. 3A). Colocalization studies revealed that in the control nerve, the highest number of RAGE-positive fibers contained CML, ∼78.88 ± 1.34%, followed by mDia1, ∼75.64 ± 3.82%, and the least stained for HMGB1 ∼41.95 ± 4.91%. In the idiopathic nerve, the highest number of RAGE-positive fibers costained for CML, ∼90.69 ± 0.4%, followed by HMGB1, ∼76.80 ± 7.38%, and mDia1, ∼66.83 ± 4.23%, while in the diabetic nerve, ∼90.18 ± 0.13% of RAGE-positive fibers stained for CML, ∼81.75 ± 2.63% stained for mDia1, and ∼73.14 ± 5.51% stained for HMGB1 (Fig. 3B).

## Discussion

Peripheral neuropathies, regardless of their etiology, share similarities in the structural and microscopic level manifestation in the damaged nerve (Donofrio 2012). Observed pathological changes are often not disease-specific and that notion prompted us to hypothesize that there might be a common molecular link underlying the pathogenesis of neuropathy. Based on our and other studies we speculate that one of such molecular links might be a key inflammation protein, RAGE. Our previous studies revealed that RAGE expression is higher in porcine (Juranek et al. 2010) and murine (Toth et al. 2008; Juranek et al. 2013) diabetic versus control nerve, contributing to the inflammatory mechanisms leading to the development and/or progression of diabetic neuropathy. It has been shown that RAGE plays a role in exacerbating existing preneuropathic or neurodegenerative conditions (Rong et al. 2005; Vicente Miranda and Outeiro 2010) by binding to its glycation or inflammatory ligands such as AGEs and triggering nuclear factor kappa-light-chain-enhancer of activated B cells (NF-κB) activation and consequently increasing neuronal stress and inflammatory responses that further damage neural structures (Takeuchi et al. 2000; Vicente Miranda and Outeiro 2010). Recently, we showed that in the postacute injury diabetic nerve, genetic deletion of *Ager* (RAGE) promotes macrophage polarization and enhances inflammatory responses leading to improved nerve regeneration scores despite hyperglycemia (Juranek et al. 2013).

In the present study, we found that in a normal, healthy human nerve, RAGE is expressed in almost 30% of all nerve fibers and that number is higher in pathological states such as peripheral neuropathy. We also found that the expression level of RAGE was higher in neuropathic nerves as compared to the control nerve. Given that the disease was already established, it is not possible to discern if such expression changes were consequences of local traumatic or toxic events and/or whether those changes might have preceded or occurred concurrently with hyperglycemia-evoked peripheral nerve changes observed in diabetes.

With respect to the ligands of RAGE in the nerve tissue, we found that the expression of two of the RAGE ligands, HMGB1 and CML, is also higher in the neuropathic nerves; however, their expression levels varied between the neuropathic specimens and controls. While the level of HMGB1 was similar in both idiopathic and diabetic neuropathic nerve, CML level was significantly different between the neuropathic specimens. The observed differences might be explained by the fact that following injury, HMGB1 is not only secreted by immune cells but by neurons as well, thus potentially promoting nerve repair by enhancing axonal sprouting and outgrowth (Lotze and Tracey 2005; Tian et al. 2009). Further, its expression might be unaffected by secondary, hyperglycemic, conditions observed in the diabetic peripheral neuropathy (Faraco et al. 2007; Shibasaki et al. 2010; Feldman et al. 2012; Juranek et al. 2013). In addition, it has been shown in vitro that blocking RAGE inhibits HMGB1-mediated neuronal development (Hori et al. 1995) indicating that RAGE-HMGB1 interaction might be crucial for repair mechanisms in the neuropathic nerve. On the contrary, CML is thought to be one of the key molecules in the inflammatory, RAGE-NF-κB-dependent pathway in diabetes, but also, independently from RAGE, it plays an important role in cumulative oxidative stress-induced neuronal changes, (Haslbeck et al. 2007), contributing to the pathogenesis of neuropathy. Extensive study of CML expression in different types of peripheral neuropathies (Haslbeck et al. 2007) revealed that increased oxidative stress and/or CML-RAGE-NF-κB-activated pathway likely plays a role in diabetes, vitamin B12 deficiency related and chronic inflammatory demyelinating peripheral neuropathy, however, the authors did not observe either RAGE or CML increase in the idiopathic neuropathy. This discrepancy might be explained by the fact that the term “idiopathic neuropathy” comprises many different, unrelated, neuropathies caused by multiple factors and cannot be treated as one disease entity.

Finally, we found that the expression of mDia1, a cytoplasmic actin-binding protein, described for the first time as an intracellular RAGE ligand in 2008 (Hudson et al. 2008), remained unaltered in idiopathic neuropathy, but was slightly lower in diabetic neuropathy. These intriguing findings suggest that further investigation is essential to address if mDia1 plays roles in human diabetic neuropathy. Perhaps the impact of mDia1 in this setting is RAGE independent; for example, these findings might suggest that mDia1 contribution to the neuropathy pathogenesis might be a result of its primary, rho-mediated cytoskeleton regulatory functions (Rose et al. 2005; Shinohara et al. 2012), and is complementary to RAGE-stimulated phosphorylation of Akt (protein kinase B) and cell proliferation/migration observed in other cell types such as smooth muscle cells (Rai et al. 2012). More detailed studies have been designed to decipher the role and expression of these proteins over long periods of time in the human peripheral nerve.
